# Introduction: the International Conference on Intelligent Biology and Medicine (ICIBM) 2016: special focus on medical informatics and big data

**DOI:** 10.1186/s12911-017-0462-0

**Published:** 2017-07-05

**Authors:** Cui Tao, Yang Gong, Hua Xu, Zhongming Zhao

**Affiliations:** University of Texas, Health Science Center at Houston, of Biomedical Informatics, Houston, TX USA

## Abstract

In this editorial, we first summarize the 2016 International Conference on Intelligent Biology and Medicine (ICIBM 2016) held on December 8–10, 2016 in Houston, Texas, USA, and then briefly introduce the ten research articles included in this supplement issue. At ICIBM 2016, a special theme, “Medical Informatics and Big Data,” was dedicated to the recent advances of data science in the medical domain. After peer review, ten articles were selected for this special issue, covering topics such as Knowledge and Data Personalization, Social Media Applications to Healthcare, Clinical Natural Language Processing, Patient Safety Analyses, and Data Mining Using Electronic Health Records.

## Introduction

The 2016 International Conference on Intelligent Biology and Medicine (ICIBM 2016) was held December 8–10, 2016 at the Texas Medical Center, Houston, Texas, USA. ICIBM 2016 included four keynote lectures, four conference invited talks, four workshops/tutorials, eight scientific sessions, and a poster session. We received 77 original manuscripts and 53 abstracts, all of which had undergone peer-review. The scientific sessions, papers and abstracts covered a great variety of research topics in bioinformatics, systems biology, big data science, biomedical informatics, pharmacogenomics, and intelligent computing. A total of 22 trainees from diverse backgrounds across the USA and international institutes were selected for travel awards and fully participated in the conference. The conference also provided a venue to allow trainees and junior investigators to meet with established scientists and, through in-depth face-to-face interactions, exchange ideas and discoveries in their research.

Biomedical science is undergoing a big data revolution. Biomedical informatics plays an essential role in the full life cycle of data science, including data harvesting, extraction, integration, normalization, and analyses. At ICIBM 2016, a special theme, “Medical Informatics and Big Data,” was dedicated to the recent advances of data science in the medical domain. It was especially attractive to conference participants, as judged from paper/abstract submission and onsite discussions during the conference. Here, we present a summary of the 10 research articles that were selected for the ICIBM 2016 special session on Medical Informatics and Big Data. All papers underwent peer-review and revision before acceptance.

### Summary of selected papers in the thematic issue

The 10 papers selected for this thematic issue were presented at ICIBM 2016. These papers cover a wide range of topics including Knowledge and Data Personalization [1, 2], Social Media Applications to Healthcare [3, 4], Clinical Natural Language Processing [5, 6], Patient Safety Analyses [7, 8], and Data Mining Using Electronic Health Records [9, 10].

As we are entering the big data era, it is important to enable data and knowledge personalization by facilitating the expression of user need, access to the relevant data based on this need, and interpretation of the data based on the user’s background [11]. Ru et al. evaluated clinician’s reading preferences regarding scientific literature, which can serve as useful information for developing a personalized literature-recommender system for clinicians who work at the front-line of patient care [1]. While Ru et al. focused on clinician users, Amith et al. focused on patients. Amith et al. developed an ontology-based approach for representing knowledge about HPV-causing cancer for patients [2]. Although focusing on cancer related knowledge, their approach can presumably be applied to any other domain for creating ontologies.

With the advent of social media and the rapidly growing volume of user-generated data on social media, there is an emergent interest in using social media data for healthcare related studies [12]. Du et al. developed a machine learning based sentiment analysis system to extract public opinions towards HPV vaccines from Twitter [3]. They further conducted time trend analyses on related tweets, which could potentially provide feedback to public health professionals to monitor online public responses, examine the effectiveness of their HPV vaccination promotion strategies, and adjust their promotion plans. Zhang et al. surveyed how the public use WeChat, the most widely and frequently used social media in China, to obtain health information [4]. This study predicted that WeChat could play on a central role in individualized health education in China.

Entity recognition is one of the most basic steps for natural language processing. Chen et al. developed a novel active learning (AL) algorithm and the first AL-enabled annotation system for clinical named entity recognition (NER) [5]. In contrast to simulation experiments, the user study here revealed that AL did not always save annotation time when compared with random sampling, indicating the need for developing querying algorithms that model the actual annotation time. In Liu et al., the authors leveraged recurrent neural network (RNN), one of the deep learning methods, for clinical entity recognition and protected health information recognition [6]. Their results show that deep learning outperforms traditional machine learning methods that suffer from fuzzy feature engineering.

Patient safety research endeavors to prevent and reduce harm to patients during the process of health care. Kang et al. developed a similarity-searching module that can dynamically measure the similarities of events in patient safety event reporting systems [7]. The similarity analysis of events is key to learning from retrospective analysis, promoting proactive identification and remediating safety events. The work presented by Cai et al. investigated the temporal variation of adverse effects of vaccines that provides a new angle for examining the data from adverse event reporting systems [8]. The application of informatics approaches to innovative design patient safety event reporting system holds promise in advancing safety event data analytics.

Exploiting the rich information in electronic health records is a mainstay of health informatics aimed at improving the quality of health care. The project detecting and quantifying new and redundant information over longitudinal clinical notes presented by Zhang et al. discussed an automated technique with statistical language models to improve data quality [9]. Kaewprag et al. [10] explored a predictive analysis of pressure ulcer in intensive care unit patient records, which helps clinicians identify relationships between risk factors associated with pressure ulcers.

## Discussion and conclusion

In the big data era, the bottleneck in biomedical research has shifted from data collection to data management and analysis. Medical informatics, an interdisciplinary field that studies the effective uses of data, information, and knowledge for solving medical problems, plays an essential role in biomedical data science. As shown in this workshop, diverse types of data including social media, clinical documents, safety reports, and patient records have been studied. Moreover, new methods and algorithms (e.g., those involving NLP and predictive modeling) have been developed to process, integrate, and analyze these data into knowledge and actionable wisdom, thus to facilitate clinical research and to improve clinical practice. We envision rapid growth of the medical informatics field and hope to organize similar future events to promote medical informatics research in the big data era.
